# TRPV4 regulates matrix stiffness and TGFβ1‐induced epithelial‐mesenchymal transition

**DOI:** 10.1111/jcmm.13972

**Published:** 2018-11-18

**Authors:** Shweta Sharma, Rishov Goswami, David X. Zhang, Shaik O. Rahaman

**Affiliations:** ^1^ Department of Nutrition and Food Science University of Maryland College Park Maryland; ^2^ Department of Medicine Medical College of Wisconsin Milwaukee Wisconsin

**Keywords:** calcium, epithelial‐mesenchymal transition, keratinocytes, matrix stiffness, TAZ, TRPV4, YAP

## Abstract

Substrate stiffness (or rigidity) of the extracellular matrix has important functions in numerous pathophysiological processes including fibrosis. Emerging data support a role for both a mechanical signal, for example, matrix stiffness, and a biochemical signal, for example, transforming growth factor β1 (TGFβ1), in epithelial‐mesenchymal transition (EMT), a process critically involved in fibrosis. Here, we report evidence showing that transient receptor potential vanilloid 4 (TRPV4), a mechanosensitive channel, is the likely mediator of EMT in response to both TGFβ1 and matrix stiffness. Specifically, we found that: (a) genetic ablation or pharmacological inhibition of TRPV4 blocked matrix stiffness and TGFβ1‐induced EMT in normal mouse primary epidermal keratinocytes (NMEKs) as determined by changes in morphology, adhesion, migration and alterations of expression of EMT markers including E‐cadherin, N‐cadherin (NCAD) and α‐smooth muscle actin (α‐SMA), and (b) TRPV4 deficiency prevented matrix stiffness‐induced EMT in NMEKs over a pathophysiological range. Intriguingly, TRPV4 deletion in mice suppressed expression of mesenchymal markers, NCAD and α‐SMA, in a bleomycin‐induced murine skin fibrosis model. Mechanistically, we found that: (a) TRPV4 was essential for the nuclear translocation of YAP/TAZ (yes‐associated protein/transcriptional coactivator with PDZ‐binding motif) in response to matrix stiffness and TGFβ1, (b) TRPV4 deletion inhibited both matrix stiffness‐ and TGFβ1‐induced expression of YAP/TAZ proteins and (c) TRPV4 deletion abrogated both matrix stiffness‐ and TGFβ1‐induced activation of AKT, but not Smad2/3, suggesting a mechanism by which TRPV4 activity regulates EMT in NMEKs. Altogether, these data identify a novel role for TRPV4 in regulating EMT.

## 1 INTRODUCTION

Substrate stiffness (or rigidity) of the extracellular matrix (ECM) has important functions in numerous physiological and pathological processes including development, wound healing, oncogenesis and tissue fibrosis.^1^, ^2^, ^3^, ^4^, ^5^, ^6^, ^7^, ^8^ Cells detect differences in matrix stiffness and adjust expression of genes and proteins in response, a capacity that has increasingly become a subject of research because of its importance in critical cellular processes including cell differentiation, migration and proliferation.^1^, ^2^, ^3^, ^4^, ^5^, ^6^, ^7^, ^8^ The epithelial‐mesenchymal transition (EMT) is a cell differentiation process by which polarized epithelial cells undergo biochemical/molecular changes, losing their cell polarity and cell‐cell adhesion and gaining migratory capacity, invasiveness and elevated resistance to apoptosis, thereby assuming a mesenchymal phenotype.^9^, ^10^, ^11^, ^12^, ^13^ EMT has essential functions in fundamental cellular processes including embryogenesis, development, tissue repair and oncogenesis.^9^, ^10^, ^11^, ^12^, ^13^ However, unchecked and exacerbated EMT can contribute to various pathological conditions such as metastasis and tissue fibrosis.^9^, ^10^, ^11^, ^12^, ^13^ Fibrotic diseases including pulmonary, liver and skin fibrosis are characterized by an increase in the invasion and migration of mesenchymal cells across a stiffened ECM associated with induction of EMT.^12^, ^13^, ^14^

Transforming growth factor β1 (TGFβ1)‐induced EMT in primary human keratinocytes and altered expression of various EMT markers in skin tissues from scleroderma, keloids and melanoma patients have recently been demonstrated, indicating a link between EMT and skin fibrosis and oncogenesis.^15^, ^16^, ^17^, ^18^, ^19^ Mechanical cues, for example, stiffness, in the ECM can influence cellular functions such as cell spreading, motility, differentiation, proliferation, and apoptosis, which are also regulated by TGFβ1.^7^, ^20^, ^21^, ^22^, ^23^ Emerging data support a role for matrix stiffness in EMT in epithelial cells.^16^, ^21^, ^22^, ^23^ Zarkoob et al^16^ have demonstrated specific effect of matrix stiffness on keratinocyte colony formation. Furthermore, it has been reported that calcium signalling and expression of specific Ca^2+^‐permeable ion channels are involved in induction of EMT.^24^, ^25^, ^26^, ^27^, ^28^ However, role of matrix stiffness and/or calcium signalling‐induced EMT in skin keratinocytes has not been reported. Although the signalling mechanisms underlying EMT have been well studied, the identity of a matrix stiffness‐sensing plasma membrane receptor/channel and the molecular mechanisms by which matrix stiffness signals are transmitted and propagated to drive EMT remain to be determined. Recently, we reported that transient receptor potential vanilloid 4 (TRPV4), a mechanosensitive Ca^2+^‐permeable channel, is associated with skin and lung fibrosis. TRPV4 regulates both biochemical (TGFβ1)‐ and mechanical (matrix stiffness) stimulus‐induced lung and dermal myofibroblast differentiation and contributes to the development of in vivo pulmonary and skin fibrosis in murine models.^29^, ^30^, ^31^ However, the specific role of TRPV4 in EMT in normal primary epidermal keratinocytes has not been determined.

Transient receptor potential vanilloid 4, a member of the TRP superfamily, is ubiquitously expressed in various cell types including skin keratinocytes and fibroblasts.^29^, ^30^, ^31^, ^32^, ^33^, ^34^ Published work by our group and others showed that TRPV4 is activated by a wide variety of physical and biochemical stimuli including changes in osmolarity, temperature, mechanical stress, UVB exposure, growth factors and metabolites of arachidonic acid.^29^, ^30^, ^31^, ^32^, ^33^, ^34^, ^35^, ^36^, ^37^, ^38^, ^39^ TRPV4 was shown to function as a mechanosensor in response to cyclical stretching of cells, mechanical forces applied to β1 integrins in cell surface or dynamic loading in chondrocytes.^34^, ^40^, ^41^, ^42^ Structurally, TRPV4 contains a number of potential regulatory domains and protein‐interaction sites, including phosphoinositide 3‐kinase (PI3K) and Src homology 2 recognition domains, putative protein kinase C phosphorylation sites, PDZ domains and an N‐terminal ankyrin repeat region.^29^, ^30^, ^31^, ^32^, ^33^, ^34^ Numerous mutations in TRPV4 have been associated with human diseases including skeletal dysplasia and sensory and motor neuropathies.^32^, ^33^ TRPV4 is linked to multiple physiological and pathological functions including sheer stress detection in blood vessels, development of joint diseases, skeletal malformations, pain sensation and osteoclast differentiation in bone.^29^, ^30^, ^31^, ^32^, ^33^, ^34^, ^35^, ^36^, ^37^, ^38^, ^39^, ^43^, ^44^, ^45^, ^46^, ^47^, ^48^ Published data from our laboratory and others showed that in mice TRPV4 deletion is associated with altered blood pressure and vasodilatory responses, vascular permeability, bladder function, lung injury and fibrosis and skin fibrosis.^29^, ^30^, ^31^, ^32^, ^33^, ^34^, ^35^, ^36^, ^37^, ^38^, ^39^, ^43^, ^44^, ^45^, ^46^, ^47^, ^48^ It has been shown that TRPV4 activation increases Ca^2+^ influx in human keratinocytes and promotes cell‐cell junction formation between these cells, whereas TRPV4 antagonism inhibits development of transepithelial resistance in cultured human keratinocytes.^38^, ^39^ However, a potential role of TRPV4 mechanosensing in EMT and the intracellular molecular pathway by which EMT signals are transmitted/propagated has not been reported.

A variety of biomarkers are associated with EMT in vitro and in vivo.^9^, ^10^, ^11^, ^12^, ^13^ For example, E‐cadherin (ECAD) is expressed in epithelial cells, and its expression is decreased during EMT.^9^, ^10^, ^11^, ^12^, ^13^ In general, loss of ECAD expression promotes EMT. The switch from ECAD to N‐cadherin (NCAD), which is expressed in mesenchymal cells, is a marker for the progress of EMT.^9^, ^10^, ^11^, ^12^, ^13^ Increased expression of α‐smooth muscle actin (α‐SMA) in myofibroblasts during EMT is also a mesenchymal marker.^9^, ^10^, ^11^, ^12^, ^13^ TGFβ1 is a well studied EMT and profibrotic mediator associated with both myofibroblast differentiation in vitro and of fibrogenesis of many organs including skin.^9^, ^10^, ^11^, ^12^, ^13^, ^15^, ^16^, ^17^, ^18^ TGFβ1 signals via a plasma membrane‐localized receptor complex of TGFβ1‐receptor type I and II resulting in downstream activation of either the transcription factors Smad2/3 pathways or through non‐Smad pathways that include c‐Jun N‐terminal kinase, p38 kinases, focal adhesion kinase, PI3K/AKT and Rho GTPases.^49^, ^50^, ^51^ Both Smad and non‐Smad pathways have been linked to EMT, myofibroblast differentiation, fibrosis and oncogenesis.^49^, ^50^, ^51^ Furthermore, transcriptional co‐activators YAP and TAZ are the major downstream effectors of the Hippo pathway, which have also been shown to induce EMT in various cell types in a context‐dependent manner.^52^, ^53^, ^54^, ^55^, ^56^ When the Hippo pathway is inactive, unphosphorylated YAP/TAZ translocate to the nucleus where they bind to a complex involving TEAD transcription factors and activate proliferative and anti‐apoptotic genes, resulting in an invasive cell phenotype.^52^, ^53^, ^54^, ^55^, ^56^ Both YAP and TAZ are mechano‐activated by stiff ECM in various cell types, and have been linked to the pathophysiology of wound healing, fibrosis and oncogenesis.^53^, ^57^, ^58^, ^59^, ^60^ Reports also suggest crosstalk between YAP/TAZ signalling and TGFβ1 signalling.^54^, ^59^, ^60^

In this study, we sought to determine the role of TRPV4 in EMT processes. We found that epidermal keratinocytes sense mechanical (stiffness) and biochemical (TGFβ1) stimuli via TRPV4 channels, and assume a mesenchymal phenotype. We also obtained in vivo evidence that TRPV4 KO mice are protected from bleomycin‐induced EMT. We found that TRPV4 deletion inhibited activation of YAP/TAZ and AKT signals in epidermal keratinocytes. Altogether, these results identify a novel regulatory role for TRPV4 in EMT induced by both TGFβ1 and pathophysiological matrix stiffness.

## 2 RESULTS

### 2.1 TRPV4 mediates TGFβ1‐induced EMT‐like changes in normal mouse primary epidermal keratinocytes

**Figure 1:**
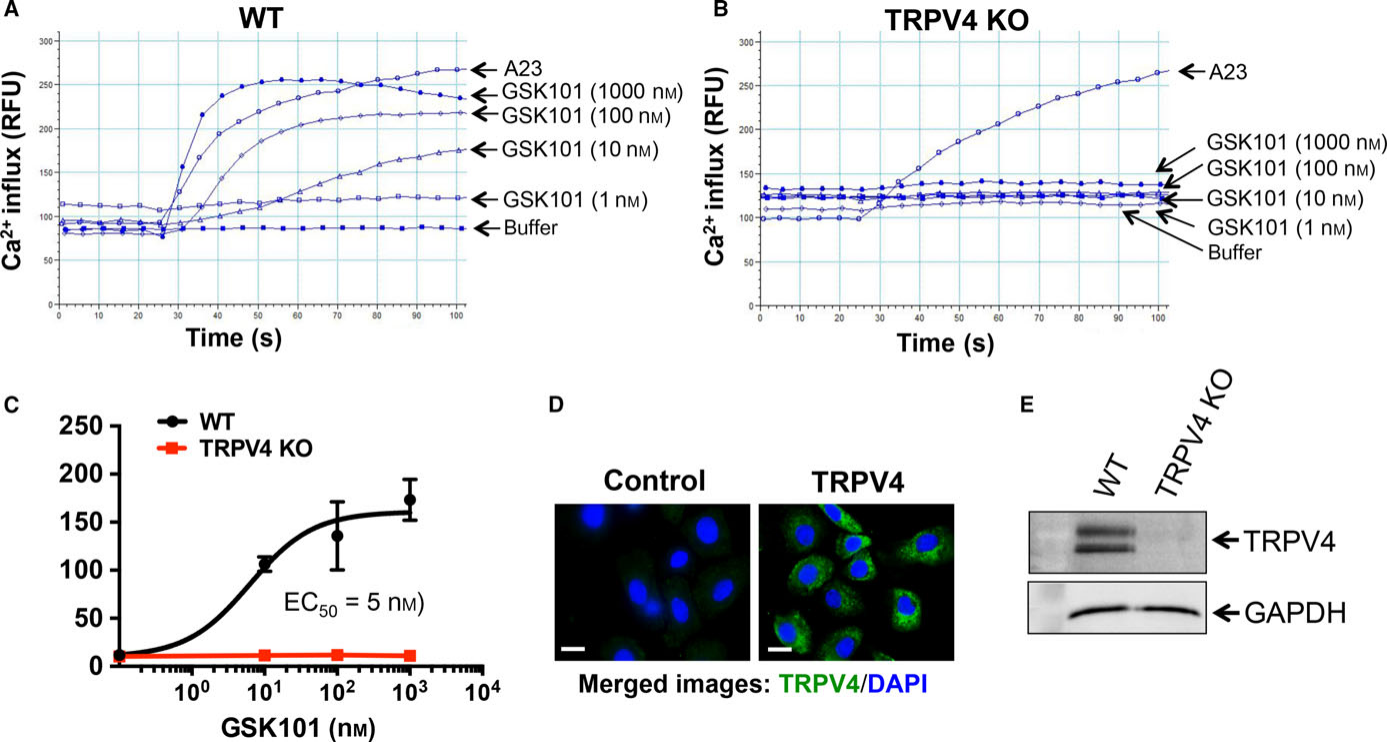
Transient receptor potential vanilloid 4 (TRPV4) Ca^2+^‐permeable channels are functional in normal primary mouse epidermal keratinocytes (NMEKs). NMEKs (15 000 cells per well) were seeded on collagen‐coated (10 μg/ml) 96‐well plastic plate. Ca^2+^ influx is shown by relative fluorescence units (RFUs) measuring ΔF/F (max‐min). A23 (2 μmol/L), a calcium ionophore, was used as a positive control. (A, B) FlexStation 3 recording of calcium 5 dye‐loaded WT and TRPV4 KO NMEK monolayers showing an effect of TRPV4 agonist GSK101 on Ca^2+^ influx. (C) Quantitation of results (mean ± SEM) from A and B. All experiments were repeated three times in quadruplicate. (D) NMEKs from WT mice were immunostained with TRPV4 antibody (green) and DAPI (blue). NMEKs pre‐incubated with TRPV4 blocking peptide (left panel) or without blocking peptide (right panel). (E) NMEKs from WT and TRPV4 KO mice were immunostained with TRPV4 antibody

To determine whether TRPV4 channels are required in EMT, we first determined the presence of functional Ca^2+^ permeable TRPV4 channels in normal mouse primary epidermal keratinocytes (NMEKs) by comparing Ca^2+^ influx in NMEKs derived from TRPV4 KO to those of wild‐type (WT) mice. We recorded a concentration‐dependent (1‐1000 nmol/L) increase in Ca^2+^ influx in WT NMEKs by a selective TRPV4 agonist, GSK101 (EC_50_ = 5 nmol/L), which was not seen in TRPV4 KO NMEKs (Figure 1A‐C).^29^, ^61^ These results confirmed the presence of functional TRPV4 calcium‐permeable channels in NMEKs, as reported previously.^38^, ^39^ Immunofluorescent and immunoblot staining indicated the presence of TRPV4 proteins in NMEKs (Figure 1D,E). Together, these results indicate that functional Ca^2+^ permeable TRPV4 channels are expressed in NMEKs.

**Figure 2:**
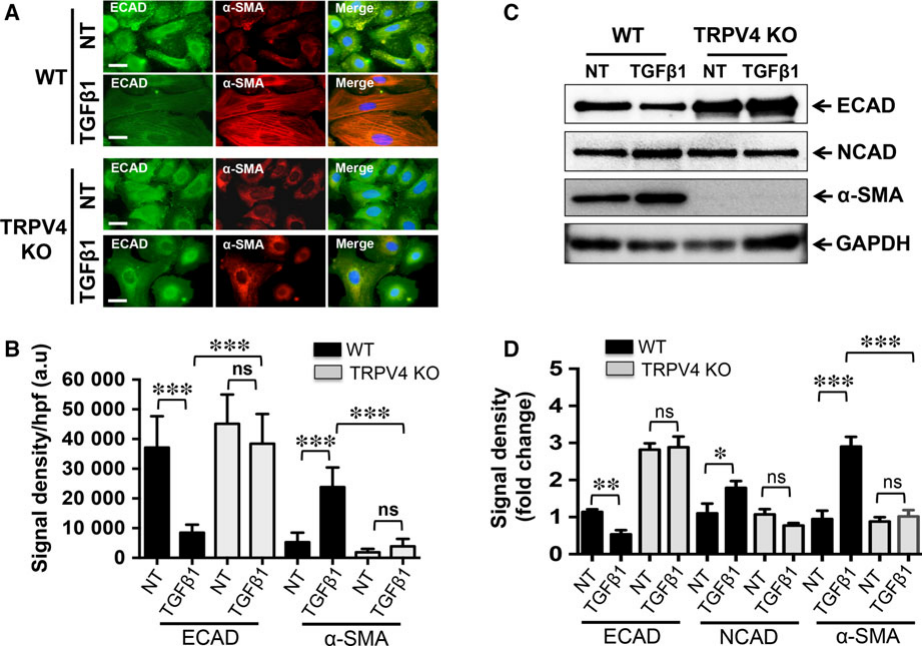
Transient receptor potential vanilloid 4 (TRPV4) is required for transforming growth factor β1 (TGFβ1)‐induced EMT‐like phenotypic/biochemical changes in normal mouse primary epidermal keratinocytes (NMEKs). NMEKs from WT and TRPV4 KO mice were plated on collagen‐coated (10 μg/ml) plastic wells and incubated with or without TGFβ1 (5 ng/ml, 72 h). (A) Representative images (from a total of five different fields per condition) showing co‐localization of ECAD (green) and α‐SMA (red) with DAPI (blue) staining in NMEKs from WT and TRPV4 KO mice. (B) Quantitation of results from (A). Data are expressed as mean ± SEM of three independent experiments; ns = non‐significant; ***P <* *0.001; n = 10 cells/condition, one‐way ANOVA. (C) Representative immunoblots from three independent experiments per condition showing an effect of TGFβ1 on ECAD, NCAD and α‐SMA protein expression in WT NMEKs and in TRPV4 KO cells. (D) Quantitation of results from (C) using GAPDH as internal control. Data are expressed as mean ± SEM, n = 3; ns = non‐significant; *P <* *0.05; **P <* *0.01; ***P <* *0.001; Student's *t* test

Transforming growth factor β1 is known to induce EMT in keratinocytes.^15^ Recent evidence from our group suggests a link between TRPV4 activation and TGFβ1 signals that mediate fibroblast differentiation.^29^, ^30^, ^31^ Previous reports have documented that calcium signalling is involved in EMT in many cell types.^24^, ^25^, ^26^, ^27^, ^28^ However, the identity of a plasma membrane calcium channel and the mechanism by which calcium signals are transduced/propagated in cells to drive EMT, are not well understood. To test our hypothesis that TRPV4 activation might regulate EMT, NMEKs from WT and TRPV4 KO mice were treated with TGFβ1, and compared for acquisition of EMT‐like phenotypic properties or EMT‐related biochemical changes. We found that compared to untreated WT controls, TGFβ1 pre‐treated WT NMEKs showed down‐regulated ECAD expression along with up‐regulated α‐SMA expression (Figure 2A,B), confirming the pro‐EMT effect of TGFβ1. Furthermore, as demonstrated by immunoblot analysis, TGFβ1 induced a decrease in ECAD, an increase in NCAD and an increase in α‐SMA expression in WT NMEKs compared to the relevant untreated controls (Figure 2C,D). In contrast, results of both immunofluorescence and immunoblot analysis indicated that TRPV4 KO NMEKs treated or not with TGFβ1 showed significant reductions in expression of α‐SMA signals compared to TGFβ1‐treated WT NMEKs (Figure 2A‐D). TGFβ1 had no significant effect on ECAD and NCAD induction in TRPV4 KO NMEKs (Figure 2A‐D). These results suggest that TRPV4 activity is required for TGFβ1‐induced EMT‐like changes in NMEKs.

### 2.2 Matrix stiffness triggers TGFβ1‐induced EMT‐like changes in NMEKs and MDFs and is dependent on TRPV4

**Figure 3:**
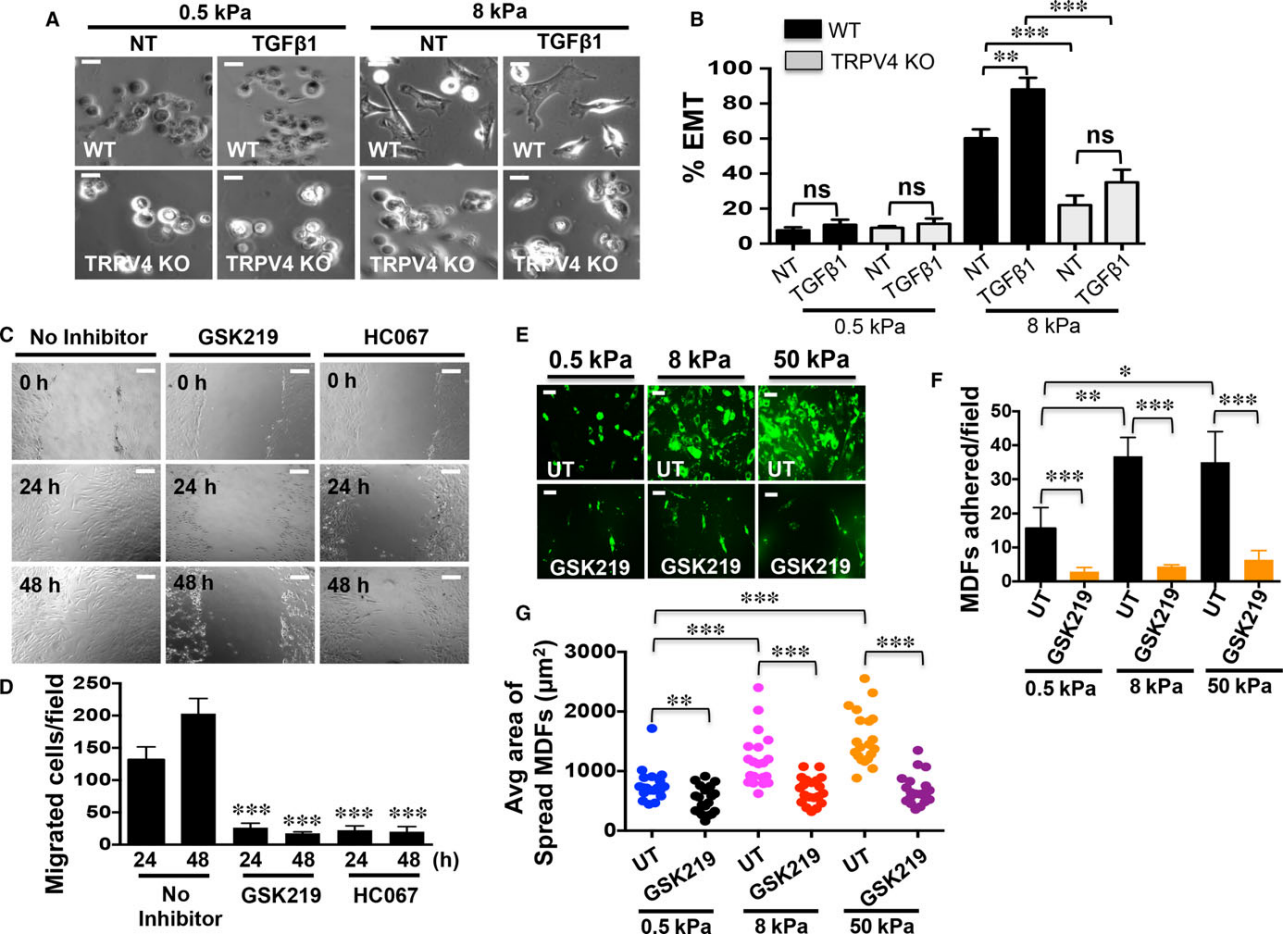
Transient receptor potential vanilloid 4 (TRPV4) regulates matrix stiffness‐induced and transforming growth factor β1 (TGFβ1)‐induced EMT‐like phenotypic and migratory/adhesive properties changes. WT and TRPV4 KO NMEKs or mouse dermal fibroblasts (MDFs) were plated on collagen‐coated compliant (0.5 kPa) and stiff (8 and 50 kPa) polyacrylamide hydrogels and incubated with or without TGFβ1 (5 ng/ml). (A) Representative images from five different fields per condition showing morphological changes in WT and TRPV4 KO NMEKs in response to increasing matrix stiffness and TGFβ1 treatment. (B) Quantitation of results from (A). Data are expressed as mean ± SEM, n = 100 cells/condition; ns = non‐significant; **P <* *0.01; ***P <* *0.001, one‐way ANOVA. (C) Representative images from five different fields per condition of scratch wound assay showing migration of MDFs in response to vehicle‐treated (untreated) and TRPV4 antagonist‐treated (GSK219 and HC067) condition at indicated time points. (D) Quantitation of results from scratch wound assay of MDFs plated on collagen‐coated (10 μg/ml) plastic wells showing inhibition of wound closure in GSK219 or HC067 pretreated MDFs vs untreated (n = 3, *t* test, ***P < 0.0001). (E) Representative images showing adhesion of MDFs plated on varied stiffness hydrogels in response to vehicle‐treated (UT) and TRPV4 antagonist‐treated (GSK219) condition. (F) Quantitation of results from adhesion assay of MDFs showing reduction of cell adhesion in GSK219 pretreated MDFs vs untreated, and soft vs stiff matrix conditions (n = 3, *t* test, *P < 0.01, **P < 0.001, ***P < 0.0001). (G) Quantitation of results from adhesion assay of MDFs showing reduction of cell spread area in GSK219 pre‐treated MDFs vs untreated, and soft vs stiff matrix conditions (n = 3, *t* test, **P < 0.005, ***P < 0.0001)

It has been reported that increases in matrix stiffness drive TGFβ1‐mediated EMT in mammary gland epithelial cells.^23^ However, the identity of a matrix stiffness sensing calcium channel and the mechanism by which calcium signals are transduced/propagated into cells to drive EMT are not known. To ascertain the role of TRPV4 on EMT in response to increasing matrix stiffness alone or in combination with TGFβ1, we seeded NMEKs and HDFs on compliant (0.5 kPa) or stiff (8 and 50 kPa) polyacrylamide hydrogels treated with or without TGFβ1, and assessed the occurrence of EMT‐like changes in TRPV4 function‐deficient and WT groups. We found that TGFβ1 was unable to drive morphological changes in WT NMEKs under compliant conditions (normal skin tissue stiffness) (Figure 3A,B). Under the conditions of stiff matrix without TGFβ1 treatment, the normal epithelial morphology of WT NMEKs changed to the elongated and spindle‐like mesenchymal morphology (Figure 3A,B). Intriguingly, the addition of TGFβ1 further augmented the matrix stiffness‐induced EMT‐like phenotypic in WT NMEKs under stiff matrix conditions (Figure 3A,B). However, stiff matrix and TGFβ1 did not induce EMT‐like morphological changes in TRPV4 KO NMEKs, and these cells retained epithelial morphology (Figure 3A,B). These results indicate that TRPV4 activity is required for both matrix stiffness and TGFβ1‐induced EMT. We further examined the migratory potential of MDFs pre‐treated with TRPV4 antagonists using scratch wound assay. The quantitation of results from scratch wound assay of MDFs showed inhibition of wound closure in GSK219 or HC067 pre‐treated MDFs vs. untreated (Figure 3C,D). We evaluated the adhesive properties between vehicle‐treated and TRPV4 antagonist‐treated MDFs in response to different matrix stiffnesses. Cells pre‐treated with TRPV4 antagonist displayed reduced adhesive properties when grown on all stiffness ranges tested, suggestion critical regulatory role of TRPV4 on cell adhesion (Figure 3E,F). As expected, cells from TRPV4 antagonist‐treated MDFs grown on polyacrylamide hydrogels (0.5, 8 and 50 kPa) displayed reduced spread area compared to untreated control (Figure 3G).

**Figure 4:**
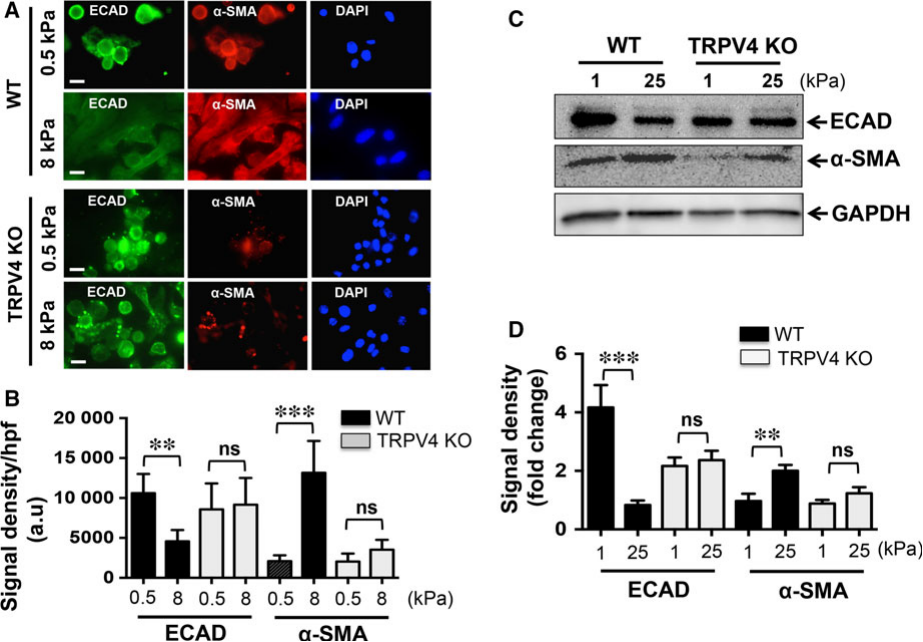
Transient receptor potential vanilloid 4 (TRPV4) deletion impairs stiffness‐induced epithelial‐mesenchymal transition in normal mouse primary epidermal keratinocytes (NMEKs). WT and TRPV4 KO NMEKs were plated on collagen‐coated compliant (0.5 kPa) and stiff (8 kPa) polyacrylamide hydrogels and incubated for 72 h. (A) Representative fluorescence images from five different fields per condition. (B) Quantitation of results from (A). Data are expressed as mean ± SEM, n = 20 cells/condition; ns = non‐significant; **P <* *0.01; ***P <* *0.001, one‐way ANOVA. (C) Representative immunoblots from three independent experiments per condition showing effect of stiffness‐induced alterations of ECAD and α‐SMA protein expression in WT NMEKs and in TRPV4 KO cells. (D) Quantitation of results from (C) using GAPDH as internal control. Data are expressed as mean ± SEM of three independent experiments; **P <* *0.01; ***P <* *0.001, *t* test

We further evaluated the status of expression of EMT markers between TRPV4 KO and WT NMEKs in response to different matrix stiffnesses by immunofluorescence staining and immunoblot. As expected, cells from WT NMEKs grown on stiff substrate (8 kPa) displayed prominent α‐SMA staining and suppressed/diffused ECAD staining (Figure 4A,B). In contrast, under similar conditions, TRPV4 KO NMEKs displayed increased ECAD staining and reduced α‐SMA staining (Figure 4A,B). No significant difference in expression of ECAD and α‐SMA was observed when both cell types were grown on soft matrix (0.5 kPa) (Figure 4A,B). Furthermore, immunoblot analysis showed that increasing stiffness induced suppression of ECAD, which was accompanied by an increase in α‐SMA levels in WT NMEKs (Figure 4C,D). However, TRPV4 KO NMEKs showed no significant change in expression levels of EMT markers under increasing stiffness conditions when compared to NMEKs grown on compliant matrix (Figure 4C,D). Altogether, these results suggest that TRPV4 is a critical regulator of both matrix stiffness and TGFβ1‐induced EMT.

### 2.3 TRPV4 deletion blocks EMT marker expression in a mouse model of bleomycin‐induced skin fibrosis

**Figure 5:**
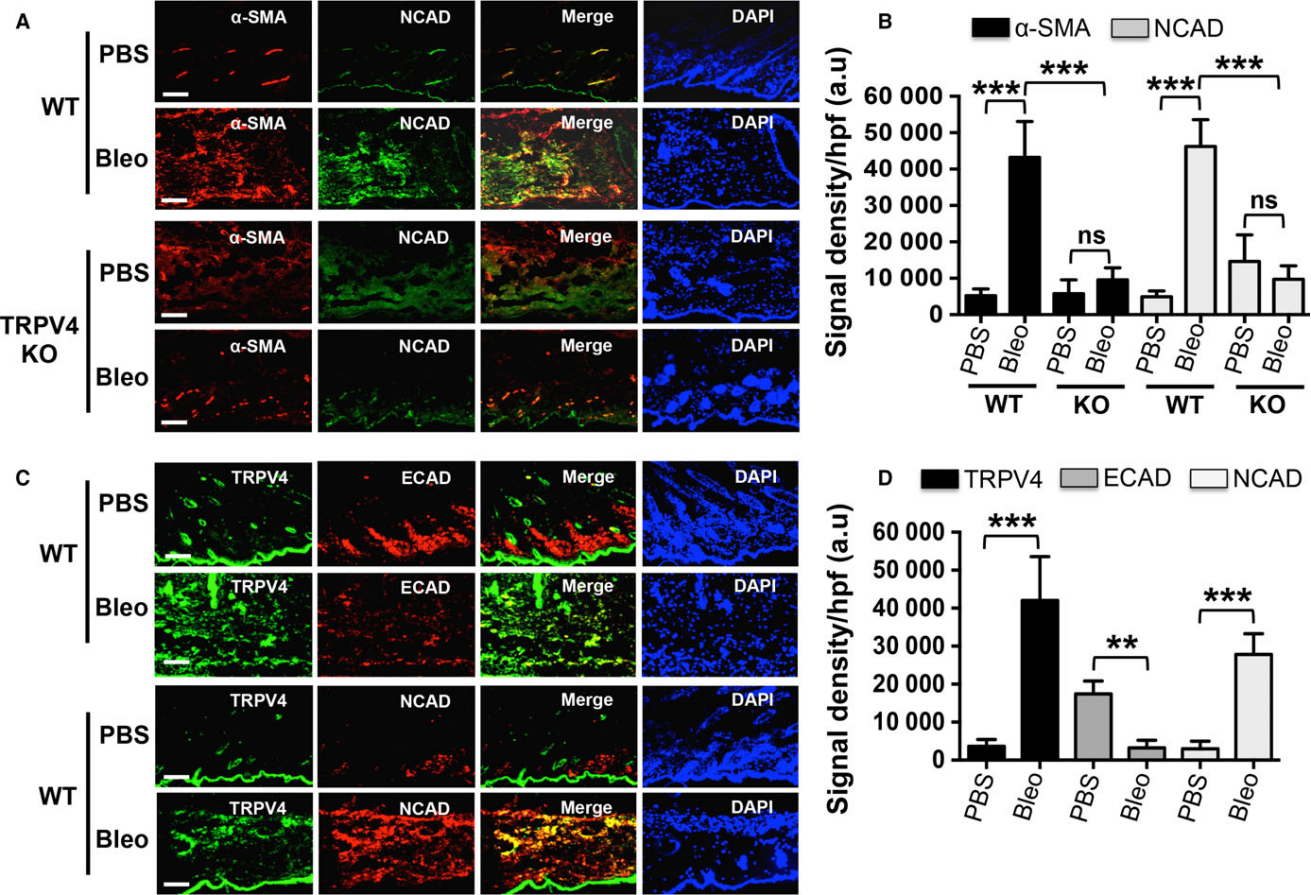
Transient receptor potential vanilloid 4 (TRPV4) deletion blocks epithelial‐mesenchymal transition (EMT) marker expression in a mouse bleomycin‐induced skin fibrosis model. WT and TRPV4 KO mice were injected subcutaneously with bleomycin (Bleo) or PBS (control) daily for 28 days, and skin sections were immunostained for α‐SMA, NCAD, ECAD and TRPV4. (A) Immunofluorescence images showing expression of α‐SMA (red) and NCAD (green) in the epidermis of Bleo‐treated WT and TRPV4 KO mice. (B) Quantitation of results from (A). Data are expressed as mean ± SEM, n = 5 mice/group; ns = non‐significant; ***P <* *0.001, one‐way ANOVA. (C) Representative immunofluorescence staining of PBS and Bleo skin sections from WT mice for TRPV4 (green) and EMT markers (ECAD and NCAD; red). Nuclei were stained with DAPI (blue). In merge, yellow colour indicates co‐localization of TRPV4 and NCAD. (D) Quantitation of results from (C). Data are expressed as mean ± SEM, n = 5 mice/group; **P <* *0.01, ***P <* *0.001, one‐way ANOVA

We previously reported that TRPV4 KO mice are protected from pro‐fibrotic effects of bleomycin in lung and skin,^29^, ^30^ and bleomycin has been shown to cause EMT in skin and pulmonary fibrosis model.^17^, ^62^ To determine whether TRPV4 deficiency could also suppress EMT in an experimental model of skin fibrosis, we employed the bleomycin‐induced skin fibrosis model, and analysed expression of EMT markers between bleomycin or phosphate‐buffered saline (PBS) treated TRPV4 KO and WT mice. Dual immunofluorescence staining of skin sections from bleomycin‐ or PBS‐treated TRPV4 KO mice showed significant reductions in the expression of mesenchymal markers (α‐SMA and NCAD) compared to control WT mice (Figure 5A,B). Furthermore, we examined the colocalization of TRPV4 with EMT markers in bleomycin treated skin tissues of fibrotic mice. TRPV4 signals in the skin tissues of bleomycin‐induced fibrotic mice were significantly increased compared to PBS‐treatment (Figure 5C,D). We found that bleomycin‐induced fibrotic skin sections from WT mice exhibited a decrease in ECAD positive and an increase in NCAD positive cells expressing TRPV4 compared to PBS‐treated controls (Figure 5C,D). These results suggest that TRPV4 may play a critical role in fibrosis in vivo by promoting EMT.

### 2.4 Matrix stiffness and TGFβ1‐induced YAP/TAZ expression and nuclear translocation are dependent on TRPV4

**Figure 6:**
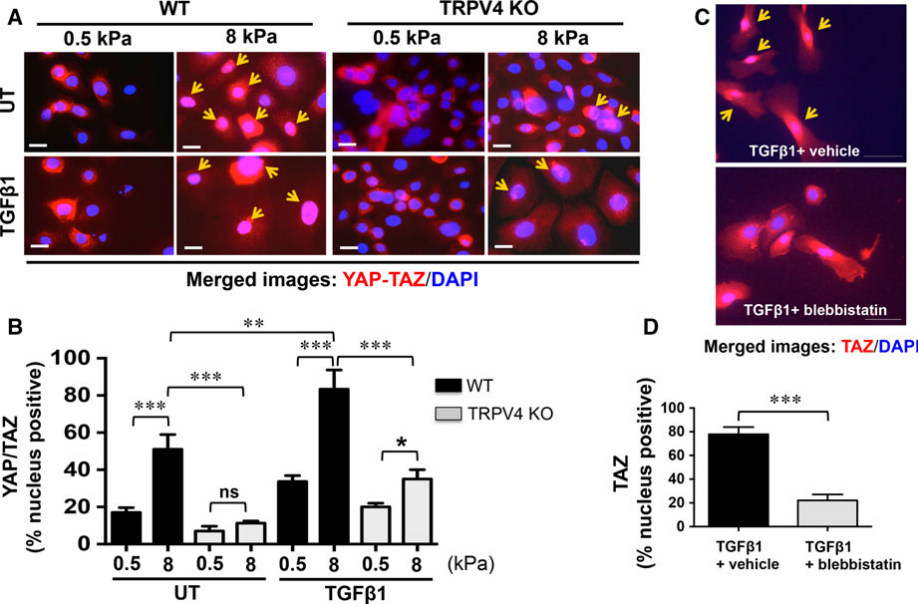
Increased stiffness potentiates transforming growth factor β1 (TGFβ1)‐induced YAP/TAZ nuclear translocation in a transient receptor potential vanilloid 4 (TRPV4)‐dependent manner. WT and TRPV4 KO NMEKs were seeded on collagen‐coated compliant (0.5 kPa) and stiff (8 kPa) polyacrylamide hydrogels and incubated with or without TGFβ1 for 72 h. Cells were stained for YAP/TAZ (red) and nuclei were stained with DAPI (blue). (A) Representative fluorescent micrographs. Yellow arrow indicates YAP/TAZ nuclear translocation. (B) Quantitation of results from (A). Data are expressed as mean ± SEM of three independent experiments; *P <* *0.05, **P <* *0.01, ***P <* *0.001, n = 20 cells/condition, one‐way ANOVA. (C) WT NMEKs were seeded on collagen‐coated plastic plates and incubated with or without blebbistatin (10 μmol/L) for 48 h to examine TGFβ1‐induced TAZ nuclear translocation. Cells were stained for TAZ (red) and nuclei were stained with DAPI (blue). (D) Quantitation of results from (C). ***P <* *0.001, n = 20 cells/condition, one‐way ANOVA

Recent evidence suggests that matrix stiffness and TGFβ1 cooperate to induce renal fibrogenesis in a YAP/TAZ‐dependent manner.^63^ Previously, it was reported that YAP and TAZ pre‐dominantly localize to the nucleus to drive EMT under high stiffness.^58^ However, the identity of a matrix stiffness sensing mechanotransduction receptor/channel and the mechanism by which mechanosensing signals are transduced/propagated into cells to drive YAP/TAZ nuclear transduction and subsequently EMT are not known. As expected, we found that stiff matrix (8 kPa) alone induced an increase in the nuclear localization of YAP and TAZ in WT NMEKs (Figure 6A,B). Pre‐treatment with TGFβ1 further amplified the stiffness‐induced YAP/TAZ nuclear accumulation in WT NMEKs compared to WT NMEKs without TGFβ1 treatment (Figure 6A,B). The absence of TRPV4 reduced the localization of YAP/TAZ to the nucleus in NMEKs grown on stiff substrate with or without TGFβ1 (Figure 6A,B). These findings suggest that TRPV4 regulates YAP and TAZ activity in response to both matrix stiffness and TGFβ1. We assessed translocation of TAZ to nucleus by immunofluorescence in WT NMEKs pre‐treated with blebbistatin (Figure 6C,D). These results indicate that blebbistatin pre‐treatment reduced TAZ nuclear translocation, suggesting TRPV4 may promote TAZ nuclear localization via cytoskeletal remodelling.

**Figure 7:**
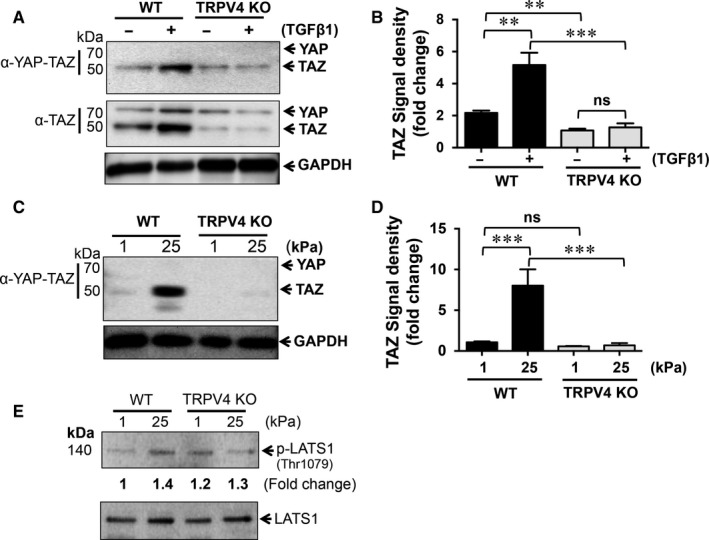
Transient receptor potential vanilloid 4 (TRPV4) deletion suppresses transforming growth factor β1 (TGFβ1) and matrix stiffness‐induced YAP/TAZ protein expression. (A) Total whole cell protein lysates from WT and TRPV4 KO NMEKs treated or not with TGFβ1 were resolved on SDS‐PAGE and immunoblotted for YAP and TAZ proteins. (B) Densitometric analysis of TAZ in immunoblots (panel A) using GAPDH as the internal control. Data are expressed as mean ± SEM of three independent experiments; **P <* *0.01; ***P <* *0.001, *t* test. (C) Representative immunoblots show effects of stiffness (1 and 25 kPa) on YAP/TAZ expression in WT and TRPV4 KO NMEKs. (D) Quantitation of results from (C). Data are expressed as mean ± SEM of three independent experiments; ns = non‐significant; ***P <* *0.001, *t* test. (E) Representative immunoblots from three independent experiments show effects of stiffness (1 and 25 kPa) on phosphorylation of Lats1 in WT and TRPV4 KO NMEKs

We further investigated the effect of matrix stiffness and TGFβ1 on the expression levels of YAP and TAZ by immunoblot analysis using two different antibodies that recognize YAP and TAZ separately. In WT NMEKs, treatment with TGFβ1 pre‐dominantly increased the expression level of TAZ compared to untreated WT controls (Figure 7A,B). The expression levels of YAP in TGFβ1‐treated WT NMEKs remained unchanged compared to untreated WT controls (Figure 7A,B). However, the absence of TRPV4 significantly decreased YAP and TAZ expression levels when compared to WT NMEKs with or without TGFβ1 (Figure 7A,B). There was no effect of TGFβ1 on YAP or TAZ expression levels between untreated and TGFβ1‐treated TRPV4 KO NMEKs (Figure 7A,B). Similarly, stiff matrix (25 kPa) caused a significant increase in TAZ expression levels in WT NMEKs, which was completely absent in TRPV4 KO NMEKs (Figure 7C,D). We also determined the impact of matrix stiffness on phosphorylation of Lats1, a Hippo pathway kinase. Immunoblot analysis show positive effects of stiffness (1 vs. 25 kPa) on phosphorylation of Lats1 in WT NMEKs; however, this effect was not dependent on TRPV4 (Figure 7E). Altogether, these findings suggest that TRPV4 is an essential regulator of YAP and TAZ expression, the critical pro‐EMT/fibrotic transcription co‐activators, in response to both matrix stiffness and TGFβ1.

### 2.5 Deletion of TRPV4 channel blocks AKT activation required for EMT

**Figure 8:**
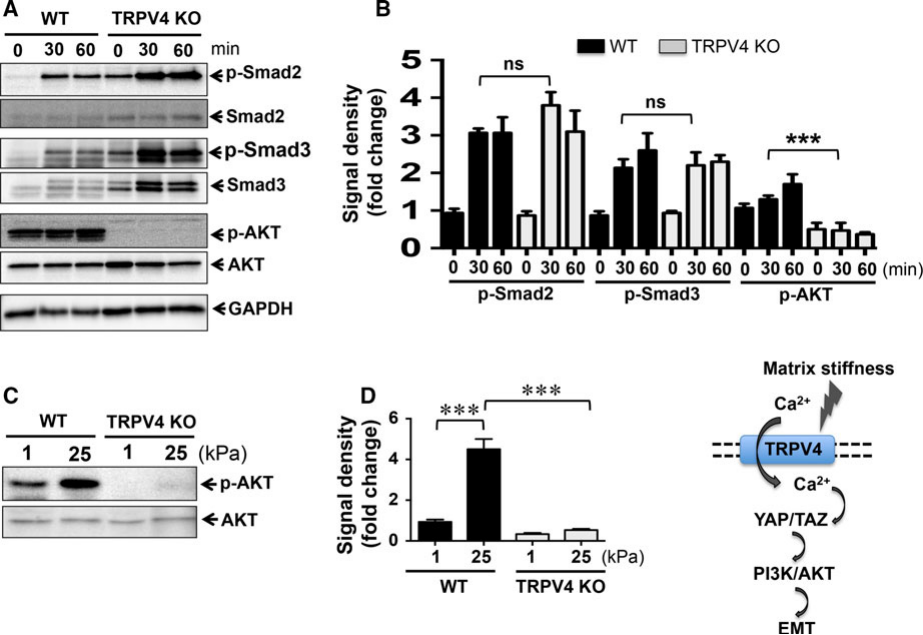
Transient receptor potential vanilloid 4 (TRPV4) deletion blocks transforming growth factor β1 (TGFβ1) and matrix stiffness‐induced AKT phosphorylation. (A) Total whole cell protein lysates were prepared from WT and TRPV4 KO NMEKs at indicated times after addition of TGFβ1 and application to matrix, and were resolved on SDS‐PAGE and immunoblotted for p‐Smad2 (at Ser 465/467), p‐Smad3 (at Ser 423/425), total Smad2, total Smad3, p‐AKT (at Ser 473) and total AKT. GAPDH was used as internal control. (B) Quantitation of results from (A). ***P <* *0.001, n = 3, *t* test. (C) Representative immunoblots to assess matrix stiffness‐induced phosphorylation of AKT in lysates from WT NMEKs and TRPV4 KO cells. (D) Quantitation of results from (C). Data are expressed as mean ± SEM of three independent experiments; ***P <* *0.001, *t* test. (E) Proposed schematic model showing mechanistic pathway by which TRPV4‐dependent calcium influx integrates matrix stiffness and soluble signals to induce EMT‐like phenomena via YAP/TAZ and PI3K/AKT pathway

Activation of the PI3K/AKT pathway is a central promoter of EMT in many cell types.^64^, ^65^ The PI3K/AKT pathway can be activated in either a TGFβ1‐dependent or ‐independent manner.^49^, ^50^, ^51^ It has been shown that activation of the PI3K/AKT pathway is up‐regulated by stiff matrices.^23^, ^66^ We found that TGFβ1‐induced activation of Smad‐2/3 proteins in a time‐dependent manner in both WT and TRPV4 KO NMEKs (Figure 8A,B). Furthermore, we found that endogenous expression and phosphorylation of Smad2/3 was increased in TRPV4 KO NMEKs treated or not with TGFβ1 compared to WT NMEKs. These results suggest that TRPV4 may act as a negative regulator of endogenous expression and phosphorylation of Smad2/3. However, genetic deletion of TRPV4 significantly suppressed phosphorylation of AKT (at serine 473) with or without TGFβ1 compared to WT NMEKs (Figure 8A,B), suggesting that both basal and TGFβ1‐induced activation of AKT are dependent on TRPV4. We compared phosphorylated AKT (p‐AKT) levels between WT and TRPV4 KO NMEKs grown on soft (1 kPa) and stiff (25 kPa) matrices. We found that stiff matrices caused increased p‐AKT levels in WT NMEKs compared to WT NMEKs grown on soft matrix (Figure 8C,D). In contrast, we found a complete absence of p‐AKT in TRPV4 KO NMEKs grown on either soft or stiff matrices (Figure 8C,D). These findings suggest that the absence of TRPV4 in NMEKs abrogates both stiffness and TGFβ1‐induced phosphorylation of AKT.

## 3 DISCUSSION

The key findings described herein are: (a) TRPV4, a mechanosensitive Ca^2+^‐permeable channel, regulates matrix stiffness and TGFβ1‐induced EMT; (b) TRPV4 deletion suppresses expression of mesenchymal markers in a murine skin fibrosis model and (c) fibrosis induced in skin tissues with bleomycin is associated with increased co‐localization of TRPV4 with mesenchymal markers and decreased co‐localization of TRPV4 with epithelial markers. We found that: (a) TRPV4 is essential for the nuclear translocation of YAP and TAZ in response to matrix stiffness and TGFβ1 in NMEKs; (b) TRPV4 deletion blocks both matrix stiffness‐induced and TGFβ1‐induced expression of YAP and TAZ proteins and (c) TRPV4 deletion abrogates matrix stiffness‐induced and TGFβ1‐induced activation of AKT, but not Smad2/3, suggesting a mechanism by which TRPV4 activity regulates EMT. These findings identify a novel role for TRPV4 in regulating EMT.

Epithelial‐mesenchymal transition has important functions in fundamental cellular and pathophysiological processes including wound healing, tissue fibrosis and oncogenesis in numerous organs such as skin, liver, kidney and lungs.^9^, ^10^, ^11^, ^12^, ^13^ Emerging data support an essential role for both a mechanical signal, for example, matrix stiffness, and a biochemical signal, for example, TGFβ1, in EMT.^16^, ^21^, ^22^, ^23^ However, the initial critical events in cell‐matrix interactions and the elicited intracellular molecular pathway by which pro‐fibrotic/EMT signals are transduced and maintained are not fully understood. It has been shown that physical and biochemical microenvironmental cues can control the degree and duration of fibrosis‐associated EMT.^22^, ^57^, ^58^, ^59^ In fibrotic tissues, the elastic modulus of normal soft tissues (0.1‐2 kPa) can reach as high as 20‐100 kPa.^8^, ^22^, ^60^, ^67^ Culturing and growing cells in a compliant or stiff matrix environment or in an environment with an elastic modulus similar to that of the normal tissue of origin, induce gene and protein expression, and thereby, produce phenotypes and functions that may not be seen when cells are cultured on plastic or glass surfaces, which are non‐physiologically stiff (elastic modulus in the range of GPa).^7^, ^20^ In this study, we studied normal mouse keratinocytes on collagen‐coated polyacrylamide hydrogels, adjusting substrate stiffness in culture, which enabled us to determine the effect of normal (compliant = 0.5‐1 kPa) or fibrotic (stiff = 8‐25 kPa) matrix on TRPV4‐dependent EMT. We found that compliant matrix was unable to cause changes in morphology or alterations of expression of EMT markers in NMEKs. In contrast, stiff matrix with or without the presence of a soluble factor (TGFβ1) induced characteristic changes associated with EMT in WT NMEKs. The presence of TGFβ1 further augmented matrix stiffness‐induced EMT‐associated changes, suggesting functional crosstalk between physical and soluble factors. These results using NMEKs are consistent with recent studies in other cell types that have demonstrated increasing matrix stiffness as a crucial regulator of TGFβ1‐induced EMT.^22^, ^23^, ^57^, ^58^, ^59^, ^60^, ^67^, ^68^, ^69^ For example, it has been shown that matrix rigidity regulates a switch between TGFβ1‐induced apoptosis and EMT in murine mammary gland epithelial cells.^23^ How matrix stiffness alone or in combination with a soluble factor like TGFβ1 modulates EMT is not well understood. Here, we found that TRPV4, a matrix stiffness‐sensitive Ca^2+^‐permeable channel, regulates matrix stiffness and TGFβ1‐induced EMT in NMEKs. These results suggest that TRPV4 is a potential mechanosensor receptor/channel involved in TGFβ1 action as an inducer during fibrogenesis and oncogenesis. These results highlight the critical role of TRPV4 in matrix stiffness‐induced EMT.

Yes‐associated protein and TAZ transcription cofactors, the key effectors of the Hippo pathway, are known to control organ size, growth, differentiation and EMT.^52^, ^53^, ^54^, ^55^, ^56^, ^57^, ^58^, ^59^, ^60^ It has been shown that YAP/TAZ control induction of fundamental cellular processes in response to matrix stiffness and TGFβ1 signalling in the context of fibrosis and oncogenesis.^52^, ^53^, ^54^, ^55^, ^56^, ^57^, ^58^, ^59^, ^60^ Published reports suggest that YAP/TAZ signalling crosstalk with TGFβ1 signalling.^54^, ^60^, ^63^, ^70^ It is well‐known that TGFβ1 binds to its receptors and induces EMT through Smad‐dependent and Smad‐independent pathways.^9^, ^49^, ^50^, ^51^, ^70^, ^71^, ^72^, ^73^ The nuclear accumulation of YAP and TAZ determines its transcriptional activity. Here, we found that TRPV4 is essential for the nuclear translocation of YAP/TAZ in response to matrix stiffness and TGFβ1 in NMEKs, suggesting a possible regulatory role of TRPV4 mechanosensing in YAP/TAZ transcriptional activity. The increased expression of YAP and TAZ proteins may modulate nuclear availability of these proteins under multiple pathophysiological conditions.^52^, ^53^, ^54^, ^55^, ^56^, ^57^, ^58^, ^59^, ^60^ Here, we found that TRPV4 deletion blocked both matrix stiffness‐induced and TGFβ1‐induced expression of YAP and TAZ proteins in NMEKs, suggesting an additional mechanism of regulating YAP/TAZ activity by TRPV4. It has been reported that matrix stiffness regulates YAP/TAZ localization to the nucleus by increasing cytoskeletal tension.^57^, ^58^ Previously, we reported that TGFβ1‐induced cytoskeletal remodelling in fibroblasts is regulated in part by TRPV4.^29^ Here, we found that TAZ nuclear localization in response to TGFβ1 is dependent on cytoskeletal remodelling. Our current results suggest that TRPV4 may promote EMT in NMEKs via cytoskeletal remodelling that regulates YAP/TAZ nuclear translocation.

We found that both WT and TRPV4 KO NMEKs were sensitive to TGFβ1‐induced Smad2/3 activation. This suggests that TRPV4 mediates EMT via a Smad‐independent pathway. However, both stiff matrices and TGFβ1‐induced activation of AKT in WT NMEKs, which was absent in TRPV4 KO NMEKs. It is known that TGFβ1‐induced EMT is regulated by activation of the PI3K/AKT pathway.^49^, ^50^, ^51^, ^71^, ^73^ It is also reported that increasing stiffness regulates TGFβ1‐induced EMT through PI3K/AKT signalling.^23^ In this study, increases in the levels of YAP/TAZ and p‐AKT correlated with the induction of EMT in WT NMEKs. Decrease in the levels of YAP/TAZ and p‐AKT correlated with the suppression of EMT in both WT and TRPV4 KO NMEKs. Together, our results suggest possible crosstalk between YAP/TAZ and AKT pathways via TRPV4 activation in driving EMT.

Bleomycin‐induced fibrosis is a well‐recognized model of fibrotic diseases including skin and lung fibrosis where matrix is stiffened in a progressive manner.^29^, ^30^, ^62^ Several lines of evidence support the role of EMT in bleomycin induced lung fibrosis.^12^, ^13^, ^62^ Recently, Zhou et al^17^ provided evidence that bleomycin induced skin epithelial cells to undergo EMT in sclerotic skin of mice. Bleomycin treatment decreased ECAD expression accompanied by increased expression of mesenchymal markers in an in vivo fibrosis model.^17^, ^62^ Our present data showed that TRPV4 is associated with bleomycin‐induced EMT. This is in agreement with our recent finding that TRPV4 deletion protects mice against bleomycin‐induced skin fibrosis.^30^

Intracellular calcium plays an important role in every aspect of cellular life including inducing EMT.^24^, ^25^, ^26^, ^27^, ^28^, ^74^, ^75^ Calcium signalling and the expression of specific Ca^2+^‐permeable ion channels are involved in induction of proteins associated with EMT.^24^, ^25^, ^26^, ^27^, ^28^ The identification of the role of specific Ca^2+^‐permeable ion channels in the induction of EMT in the context of fibrosis and oncogenesis suggests that these channels may be appropriate therapeutic targets for control of EMT‐mediated disease progression. Our recently published reports suggested that TRPV4‐dependent Ca^2+^ influx potentiates TGFβ1‐induced lung and dermal myofibroblast differentiation in response to matrix stiffness, and showed that TRPV4 KO mice were protected from bleomycin‐induced fibrosis in skin and lungs.^29^, ^30^, ^31^ Although TGFβ1 and matrix stiffness were shown to play important roles in EMT and fibrosis, the role of TRPV4 mechanosensing in EMT has not been reported. Our data are consistent with a model (Figure 8E) in which TRPV4‐dependent Ca^2+^ influx integrates matrix stiffness and soluble signals to promote EMT via YAP/TAZ and PI3K/AKT pathways, and thus may contribute to development of fibrosis and oncogenesis. However, our results warrant further studies to determine whether AKT activation is downstream to YAP/TAZ pathway. It has been reported that matrix stiffness regulates YAP/TAZ translocation to nucleus by increasing cytoskeletal tension, and the activation of Hippo pathway and the subcellular distribution of YAP is also influenced by cell attachment status.^76^ In our model we found that matrix stiffness‐induced phosphorylation of Lats1 is not dependent on TRPV4, suggesting this channel has no direct role in regulating Lats1 activation.

It is also unknown how TRPV4 is sensitized by matrix stiffness or TGFβ1. In view of the fact that TRPV4 mediates actin polymerization and integrin signalling,^29^, ^40^, ^41^ it will be interesting to determine if TRPV4 is sensitized through cytoskeletal remodelling and/or integrin signalling via a feed‐forward mechanism. In summary, we report a novel role of TRPV4 channels in regulating matrix stiffness‐induced and TGFβ1‐induced EMT in normal skin epithelial cells. Furthermore understanding the mechanism by which TRPV4 regulates EMT will support therapeutic targeting of TRPV4 signalling to combat fibrosis, foreign body response and oncogenesis.

## 4 MATERIALS AND METHODS

### 4.1 Reagents

Anti‐α‐SMA, GSK2193874 (GSK219), GSK1016790A (GSK101), A23187 (A23; calcium ionophore) were purchased from Sigma‐Aldrich (St. Louis, MO, USA). Anti‐TRPV4 primary antibody was purchased from Alomone Labs (Jerusalem, Israel), and anti‐GAPDH antibodies were purchased from Santa Cruz Biotechnology (Dallas, TX, USA). Mouse and rabbit anti‐goat IgG were purchased from Jackson Immuno‐Research (West Grove, PA, USA). Anti‐ECAD, anti‐NCAD, anti‐YAP/TAZ, anti‐YAP, anti‐TAZ, anti‐AKT, anti‐phospho‐AKT (anti‐p‐Ser473 AKT), anti‐Smad2, anti‐Smad3, anti‐p‐Smad2, and anti‐p‐Smad3 antibodies were purchased from Cell Signaling Technology (Beverly, MA, USA). TGFβ1 was purchased from R&D Systems (Minneapolis, MN, USA). Alexa Fluor 488/594 conjugated IgG and Prolong diamond DAPI were purchased from Thermo Fisher Scientific (Waltham, MA, USA). FLIPR Calcium 5 assay kit was purchased from Molecular devices (Sunnyvale, CA, USA). Easy coat polyacrylamide hydrogels of various degrees of stiffnesses (0.5, 1, 8, and 25 kPa) were purchased from Matrigen Life Technologies (Brea, CA, USA). Immunoblotting‐related reagents were purchased from Bio‐Rad Laboratories (Hercules, CA, USA). All other chemicals were purchased from Sigma or Thermo Fisher Scientific unless otherwise indicated.

### 4.2 Animals

Trpv4 knock out (TRPV4 KO or TRPV4−/−) mice on a C57BL/6 background were originally generated by Dr. M. Suzuki, Department of Pharmacology, Jichi Medical University, Tochigi, Japan.^77^ Wild‐type C57BL/6 congenic mice were purchased from Charles River Laboratories (Wilmington, MA, USA). We acquired TRPV4 KO mice from Dr. David. X. Zhang, Department of Medicine, Medical College of Wisconsin, Milwaukee, WI.^78^ All experiments on mice were performed in accordance with the Institutional Animal Care and Use Committee (IACUC) guidelines, and were approved by the University of Maryland‐College Park Review Committee. Mice were housed under pathogen‐free conditions with controlled temperature and humidity, and with food and water available ad libitum.

### 4.3 Cell culture

Primary normal mouse epidermal keratinocytes were derived from the tail of 8–10‐week‐old WT and TRPV4 KO adult mice as published previously.^79^ Briefly, the mouse tail skin was washed with sterile cold PBS, and treated with trypsin (0.25%) for 2 hours at 37°C, 5% CO_2_ to separate epidermis and dermis. The peeled epidermis was rinsed thoroughly in PBS and minced in cold keratinocyte specific media (ATCC, Manassas, VA, USA). The cell suspension was pipetted up and down several times, strained, centrifuged and resuspended in media. NMEKs were then seeded in collagen‐coated (10 μg/ml) culture flasks. Medium was replaced every 48 hours thereafter. NMEKs were maintained in complete keratinocyte media supplemented with keratinocyte growth kit (ATCC) and penicillin/streptomycin (Thermo Fisher Scientific). Cells were cultured at 37°C in a humidified atmosphere containing 5% CO_2_ and sub‐cultured at 70% confluence. Purity of NMEKs was examined by changes in morphology and by analysing alterations of expression of epithelial/keratinocyte specific markers as previously published.^9^ For all experiments, NMEKs were seeded on collagen coated (10 μg/ml) plastic or polyacrylamide hydrogels with compliant (0.5 and 1 kPa) and stiff (8 and 25 kPa) matrices followed by treatment with vehicle or TGFβ1 (5 ng/ml).

### 4.4 Bleomycin‐induced murine skin fibrosis model

The bleomycin‐induced skin fibrosis model was employed as described previously.^29^, ^30^ Bleomycin was prepared by dissolving bleomycin sulfate (Hospira, Lake Forest, IL, USA) in sterile PBS. Briefly, 6‐month‐old WT and TRPV4 KO mice (n = 5 per group) received equal volumes (0.1 ml) of bleomycin (10 mg/kg) or PBS (control). All injections were administered subcutaneously to the shaved dorsal area of the mice every alternate day for 28 days. All mice were euthanized 24 hours after the last dose, and skin tissues were harvested for immunofluorescence staining. Skin samples were snap frozen in liquid nitrogen, embedded in OCT (Sakura Finetek, USA), and stored at −80°C. Cryostat sections (7 μm) were mounted on glass slides.

### 4.5 Intracellular calcium influx assay

Changes in intracellular calcium (Ca^2+^) or calcium influx in NMEKs was performed on the FlexStation3 system using a FLIPR calcium 5 Assay Kit as previously described.^29^ Briefly, NMEKs (15 000 cells/well in complete keratinocyte media) were treated in 96‐well plates with or without TGFβ1 at 37°C, 5% CO_2_. After 24 hours, cells were incubated with FLIPR kit reagents (calcium 5 dye in 1X HBSS solution containing 20 mmol/L HEPES and 2.5 mmol/L probenecid) for 45 minutes at 37°C, followed by incubation with vehicle or TRPV4 antagonist GSK2193874 (GSK219) for 45 minutes at 37°C.^80^ Plates were incubated in the dark for another 10 minutes and then transferred to the FlexStation3 system to measure fluorescence. Calcium influx was induced by the TRPV4 agonist GSK1016790A (GSK101)^61^ in vehicle‐pretreated or GSK219‐pretreated NMEKs, and cytosolic Ca^2+^ influx was recorded by measuring ΔF/F (Max‐Min) as described previously.^29^, ^31^ Data are shown as relative fluorescence units.

### 4.6 Scratch wound healing assay and cell spreading

For scratch wound healing assay, wounded HDF monolayers were incubated in 1% BSA containing serum‐free medium with or without the indicated antagonists. Images of the wounds were captured at 0.5 hours (time 0) and 24 or 48 hours later, and the total number of migrated cells in the “wound” areas was counted. To assess morphological changes in WT, TRPV4 KO NMEKs and TRPV4 antagonist‐treated MDFs, cells were seeded on collagen‐coated polyacrylamide hydrogels with compliant (0.5 and 1 kPa) or stiff (8, 25, and 50 kPa) matrices. Cells were incubated with TGFβ1, antagonists or vehicle in complete keratinocyte or MDF media, for 72 hours. Cells were examined by phase contrast or fluorescence microscopy (Carl Zeiss, Germany) for phenotypic changes related to EMT. Images were captured, and percent EMT, cell adherence and spread area were calculated.

### 4.7 Immunofluorescence staining

Normal mouse primary epidermal keratinocytes were grown on collagen coated cover glass or polyacrylamide hydrogels, and treated with or without TGFβ1 for 72 hours. Cells were fixed with 3% paraformaldehyde, permeabilized with 0.1% Triton X‐100, washed and blocked with 3% BSA/PBS. Cells were immunostained for ECAD, α‐SMA, TRPV4, YAP and TAZ. Bleomycin or vehicle treated mouse skin sections were fixed with acetone, and immunostained for α‐SMA, NCAD, ECAD and TRPV4, followed by incubation with Alexa Fluor 488 or 594 conjugated IgG. Cells were then mounted using Prolong diamond antifade reagent with DAPI. Immunofluorescence intensity was quantified using ImageJ software (NIH), and the results were expressed as integrated density (int. density) (the product of area and mean grey value).

### 4.8 Immunoblot analysis

Normal mouse primary epidermal keratinocytes were grown on collagen‐coated tissue culture plates, treated with TGFβ1 or vehicle for 72 hours and harvested for preparation of whole cell lysate. In additional experiments, NMEKs were grown on compliant and stiff matrices, and were harvested by digesting in modified RIPA lysis buffer with protease and phosphatase inhibitors. Whole cell lysates were separated on SDS‐polyacrylamide gels and transferred to polyvinylidene difluoride membranes. Membranes were incubated with primary antibodies: anti‐ECAD (1:5000), anti‐NCAD (1:4000), anti‐α‐SMA (1:20 000), anti‐YAP/TAZ (1:1000), anti‐YAP (1:1000), anti‐TAZ (1:1000), anti‐Smad‐2/3 (1:2000), anti‐p‐Smad‐2/3 (1:1000), anti‐AKT (1:2000), anti‐p‐AKT (1:1000), anti‐GAPDH (1:3000) followed by incubation with secondary antibodies (1:5000). Immunoreactive bands were visualized using an enhanced chemiluminescence system (UVP Biospectrum, Upland, CA). The results were analysed using ImageJ and expressed as signal density. GAPDH band density was used as a loading control.

### 4.9 Statistical analysis

All data are expressed as mean ± SEM. Statistical analysis was performed with the Student's *t* test for two groups or ANOVA for three or more groups using Prism software. A value of *P* ≤ 0.05 was considered statistically significant.

## AUTHOR CONTRIBUTIONS

SS and SOR conceived the study, designed and performed the experiments and wrote the manuscript. RG assisted with experiments and analysis of data, and maintained the animal colony. DXZ helped in experimental design and interpretation. All authors reviewed the results and approved the final content of the manuscript.

## ACKNOWLEDGEMENTS AND FUNDING

Startup grant from University of Maryland, NIH (1R01EB024556‐01) and NSF (CMMI‐1662776) grants to Shaik O. Rahaman.

## CONFLICTS OF INTEREST

The authors declare that there are no conflicts of interest with the contents of this article.

## References

[1] Kumar S . Cellular mechanotransduction: stiffness does matter. Nat Mater. 2014;13:918‐920. 25241671 10.1038/nmat4094

[2] Plotnikov SV , Pasapera AM , Sabass B , Waterman CM . Force fluctuations within focal adhesions mediate ECM‐rigidity sensing to guide directed cell migration. Cell. 2012;151:1513‐1527. 23260139 10.1016/j.cell.2012.11.034 PMC3821979

[3] Sunyer R , Conte V , Escribano J , et al. Collective cell durotaxis emerges from long‐range intercellular force transmission. Science. 2016;353:1157‐1161. 27609894 10.1126/science.aaf7119

[4] Tschumperlin DJ . Fibroblasts and the ground they walk on. Physiology (Bethesda). 2013;28:380‐390. 24186933 10.1152/physiol.00024.2013 PMC3858213

[5] Irianto J , Pfeifer CR , Xia Y , Discher DE . SnapShot: mechanosensing matrix. Cell. 2016;165:1820‐1820. 27315485 10.1016/j.cell.2016.06.002 PMC5341690

[6] Nagelkerke A , Bussink J , Rowan AE , Span PN . The mechanical microenvironment in cancer: How physics affects tumours. Semin Cancer Biol. 2015;35:62‐70. 26343578 10.1016/j.semcancer.2015.09.001

[7] Yeung T , Georges PC , Flanagan LA , et al. Effects of substrate stiffness on cell morphology, cytoskeletal structure, and adhesion. Cell Motil Cytoskeleton. 2005;60:24‐34. 15573414 10.1002/cm.20041

[8] Discher DE , Janmey P , Wang YL . Tissue cells feel and respond to the stiffness of their substrate. Science. 2005;310:1139‐1143. 16293750 10.1126/science.1116995

[9] Lamouille S , Xu J , Derynck R . Molecular mechanisms of epithelial‐mesenchymal transition. Nat Rev Mol Cell Biol. 2014;15:178‐196. 24556840 10.1038/nrm3758 PMC4240281

[10] Krainock M , Toubat O , Danopoulos S , Beckham A , Warburton D , Kim R . Epicardial epithelial‐to‐mesenchymal transition in heart development and disease. J Clin Med. 2016;5:27. 10.3390/jcm5020027 PMC4773783 26907357

[11] Trimboli AJ , Fukino K , de Bruin A , et al. Direct evidence for epithelial‐mesenchymal transitions in breast cancer. Cancer Res. 2008;68:937‐945. 18245497 10.1158/0008-5472.CAN-07-2148

[12] Stone RC , Pastar I , Ojeh N , et al. Epithelial‐mesenchymal transition in tissue repair and fibrosis. Cell Tissue Res. 2016;365:495‐506. 27461257 10.1007/s00441-016-2464-0 PMC5011038

[13] Tanjore H , Xu XC , Polosukhin VV , et al. Contribution of epithelial‐derived fibroblasts to bleomycin‐induced lung fibrosis. Am J Respir Crit Care Med. 2009;180:657‐665. 19556518 10.1164/rccm.200903-0322OC PMC2753790

[14] Hinz B , Phan SH , Thannickal VJ , Galli A , Bochaton‐Piallat ML , Gabbiani G . The myofibroblast: one function, multiple origins. Am J Pathol. 2007;170:1807‐1816. 17525249 10.2353/ajpath.2007.070112 PMC1899462

[15] O'Kane D , Jackson MV , Kissenpfennig A , et al. SMAD inhibition attenuates epithelial to mesenchymal transition by primary keratinocytes in vitro. Exp Dermatol. 2014;23:497‐503. 24848428 10.1111/exd.12452

[16] Zarkoob H , Bodduluri S , Ponnaluri SV , Selby JC , Sander EA . Substrate stiffness affects human keratinocyte colony formation. Cell Mol Bioeng. 2015;8:32‐50. 26019727 10.1007/s12195-015-0377-8 PMC4442095

[17] Zhou CF , Zhou DC , Zhang JX , et al. Bleomycin‐induced epithelial‐mesenchymal transition in sclerotic skin of mice: possible role of oxidative stress in the pathogenesis. Toxicol Appl Pharmacol. 2014;277:250‐258. 24726524 10.1016/j.taap.2014.03.024

[18] Nikitorowicz‐Buniak J , Denton CP , Abraham D , Stratton R . Partially evoked epithelial‐mesenchymal transition (EMT) is associated with increased TGFβ signaling within lesional scleroderma skin. PLoS ONE. 2015;10:e0134092. 26217927 10.1371/journal.pone.0134092 PMC4517793

[19] Mirantes C , Espinosa I , Ferrer I , Dolcet X , Prat J , Matias‐Guiu X . Epithelial‐to‐mesenchymal transition and stem cells in endometrial cancer. Hum Pathol. 2013;44:1973‐1981. 23845467 10.1016/j.humpath.2013.04.009

[20] Mih JD , Sharif AS , Liu F , Marinkovic A , Symer MM , Tschumperlin DJ . A multiwell platform for studying stiffness‐dependent cell biology. PLoS ONE. 2011;6:e19929. 21637769 10.1371/journal.pone.0019929 PMC3103526

[21] Nasrollahi S , Pathak A . Topographic confinement of epithelial clusters induces epithelial‐to‐mesenchymal transition in compliant matrices. Sci Rep. 2016;6:18831. 26728047 10.1038/srep18831 PMC4700414

[22] Brown AC , Fiore VF , Sulchek TA , Barker TH . Physical and chemical microenvironmental cues orthogonally control the degree and duration of fibrosis‐associated epithelial‐to‐mesenchymal transitions. J Pathol. 2013;229:25‐35. 23018598 10.1002/path.4114 PMC5679001

[23] Leight JL , Wozniak MA , Chen S , Lynch ML , Chen CS . Matrix rigidity regulates a switch between TGF‐β1‐induced apoptosis and epithelial‐mesenchymal transition. Mol Biol Cell. 2012;23:781‐791. 22238361 10.1091/mbc.E11-06-0537 PMC3290638

[24] Davis FM , Azimi I , Faville RA , et al. Induction of epithelial‐mesenchymal transition (EMT) in breast cancer cells is calcium signal dependent. Oncogene. 2014;33:2307‐2316. 23686305 10.1038/onc.2013.187 PMC3917976

[25] Garriock RJ , Krieg PA . Wnt11‐R signaling regulates a calcium sensitive EMT event essential for dorsal fin development of Xenopus. Dev Biol. 2007;304:127‐140. 17240368 10.1016/j.ydbio.2006.12.020 PMC1905145

[26] Wu Y , Xu X , Ma L , Yi Q , Sun W , Tang L . Calreticulin regulates TGF‐β1‐induced epithelial mesenchymal transition through modulating Smad signaling and calcium signaling. Int J Biochem Cell Biol. 2017;90:103‐113. 28778674 10.1016/j.biocel.2017.07.023

[27] Stewart TA , Azimi I , Thompson EW , Roberts‐Thomson SJ , Monteith GR . A role for calcium in the regulation of ATP‐binding cassette, sub‐family C, member 3 (ABCC3) gene expression in a model of epidermal growth factor‐mediated breast cancer epithelial‐mesenchymal transition. Biochem Biophys Res Commun. 2015;458:509‐514. 25666946 10.1016/j.bbrc.2015.01.141

[28] Schaar A , Sukumaran P , Sun Y , Dhasarathy A , Singh BB . TRPC1‐STIM1 activation modulates transforming growth factor β‐induced epithelial‐to‐mesenchymal transition. Oncotarget. 2016;7:80554‐80567. 27793015 10.18632/oncotarget.12895 PMC5348340

[29] Rahaman SO , Grove LM , Paruchuri S , et al. TRPV4 mediates myofibroblast differentiation and pulmonary fibrosis in mice. J Clin Invest. 2014;124:5225‐5238. 25365224 10.1172/JCI75331 PMC4348970

[30] Goswami R , Cohen J , Sharma S , et al. TRPV4 ion channel is associated with scleroderma. J Invest Dermatol. 2017;137:962‐965. 27889423 10.1016/j.jid.2016.10.045 PMC9936819

[31] Sharma S , Goswami R , Merth M , et al. TRPV4 ion channel is a novel regulator of dermal myofibroblast differentiation. Am J Physiol Cell Physiol. 2017;312:C562‐C572. 28249987 10.1152/ajpcell.00187.2016 PMC6105932

[32] Garcia‐Elias A , Mrkonjić S , Jung C , Pardo‐Pastor C , Vicente R , Valverde MA . The TRPV4 channel. Handb Exp Pharmacol. 2014;222:293‐319. 24756711 10.1007/978-3-642-54215-2_12

[33] Everaerts W , Nilius B , Owsianik G . The vanilloid transient receptor potential channel TRPV4: from structure to disease. Prog Biophys Mol Biol. 2010; 103: 2‐17. 19835908 10.1016/j.pbiomolbio.2009.10.002

[34] Liedtke W , Tobin DM , Bargmann CI , Friedman JM . Mammalian TRPV4 (VR‐OAC) directs behavioral responses to osmotic and mechanical stimuli in *Caenorhabditis elegans* . Proc Natl Acad Sci USA. 2003;100(Suppl 2):14531‐14536. 14581619 10.1073/pnas.2235619100 PMC304114

[35] Güler AD , Lee H , Iida T , Shimizu I , Tominaga M , Caterina M . Heat‐evoked activation of the ion channel, TRPV4. J Neurosci. 2002;22:6408‐6414. 12151520 10.1523/JNEUROSCI.22-15-06408.2002 PMC6758176

[36] Vriens J , Watanabe H , Janssens A , Droogmans G , Voets T , Nilius B . Cell swelling, heat, and chemical agonists use distinct pathways for the activation of the cation channel TRPV4. Proc Natl Acad Sci USA. 2004;101:396‐401. 14691263 10.1073/pnas.0303329101 PMC314196

[37] Moore C , Cevikbas F , Pasolli HA , et al. UVB radiation generates sunburn pain and affects skin by activating epidermal TRPV4 ion channels and triggering endothelin‐1 signaling. Proc Natl Acad Sci USA. 2013;110:E3225‐E3234. 23929777 10.1073/pnas.1312933110 PMC3752269

[38] Kida N , Sokabe T , Kashio M , et al. Importance of transient receptor potential vanilloid 4 (TRPV4) in epidermal barrier function in human skin keratinocytes. Pflugers Arch. 2012;463:715‐725. 22374181 10.1007/s00424-012-1081-3

[39] Chen Y , Fang Q , Wang Z , et al. Transient receptor potential vanilloid 4 ion channel functions as a pruriceptor in epidermal keratinocytes to evoke histaminergic itch. J Biol Chem. 2016;291:10252‐10262. 26961876 10.1074/jbc.M116.716464 PMC4858974

[40] Matthews BD , Thodeti CK , Tytell JD , Mammoto A , Overby DR , Ingber DE . Ultra‐rapid activation of TRPV4 ion channels by mechanical forces applied to cell surface beta1 integrins. Integr Biol (Camb). 2010;2:435‐442. 20725677 10.1039/c0ib00034e PMC3147167

[41] Thodeti CK , Matthews B , Ravi A , et al. TRPV4 channels mediate cyclic strain‐induced endothelial cell reorientation through integrin‐to‐integrin signaling. Circ Res. 2009;104:1123‐1130. 19359599 10.1161/CIRCRESAHA.108.192930 PMC2754067

[42] O'Conor CJ , Leddy HA , Benefield HC , Liedtke WB , Guilak F . TRPV4‐mediated mechanotransduction regulates the metabolic response of chondrocytes to dynamic loading. Proc Natl Acad Sci USA. 2014;111:1316‐1121. 24474754 10.1073/pnas.1319569111 PMC3910592

[43] Köhler R , Heyken WT , Heinau P , et al. Evidence for a functional role of endothelial transient receptor potential V4 in shear stress‐induced vasodilatation. Arterioscler Thromb Vasc Biol. 2006;26:1495‐1502. 16675722 10.1161/01.ATV.0000225698.36212.6a

[44] Lamandé SR , Yuan Y , Gresshoff IL , et al. Mutations in TRPV4 cause an inherited arthropathy of hands and feet. Nat Genet. 2011;43:1142‐1146. 21964574 10.1038/ng.945

[45] Leddy HA , McNulty AL , Rothfusz NE , et al. Follistatin in chondrocytes: the link between TRPV4 channelopathies and skeletal malformations. FASEB J. 2014;28:2525‐2537. 24577120 10.1096/fj.13-245936 PMC4021446

[46] Liedtke W , Friedman JM . Abnormal osmotic regulation in trpv4‐/‐ mice. Proc Natl Acad Sci USA. 2003;100:13698‐13703. 14581612 10.1073/pnas.1735416100 PMC263876

[47] Masuyama R , Vriens J , Voets T , et al. TRPV4‐mediated calcium influx regulates terminal differentiation of osteoclasts. Cell Metab. 2008;8:257‐265. 18762026 10.1016/j.cmet.2008.08.002

[48] Muramatsu S , Wakabayashi M , Ohno T , et al. Functional gene screening system identified TRPV4 as a regulator of chondrogenic differentiation. J Biol Chem. 2007;282:32158‐32167. 17804410 10.1074/jbc.M706158200

[49] Biernacka A , Dobaczewski M , Frangogiannis NG . TGF‐β signaling in fibrosis. Growth Factors. 2011;29:196‐202. 21740331 10.3109/08977194.2011.595714 PMC4408550

[50] Akhurst RJ , Hata A . Targeting the TGFβ signalling pathway in disease. Nat Rev Drug Discov. 2012;11:790‐811. 23000686 10.1038/nrd3810 PMC3520610

[51] Wynn TA , Ramalingam TR . Mechanisms of fibrosis: therapeutic translation for fibrotic disease. Nat Med. 2012;18:1028‐1040. 22772564 10.1038/nm.2807 PMC3405917

[52] Piccolo S , Dupont S , Cordenonsi M . The biology of YAP/TAZ: hippo signaling and beyond. Physiol Rev. 2014;94:1287‐1312. 25287865 10.1152/physrev.00005.2014

[53] Low BC , Pan CQ , Shivashankar GV , Bershadsky A , Sudol M , Sheetz M . YAP/TAZ as mechanosensors and mechanotransducers in regulating organ size and tumor growth. FEBS Lett. 2014;588:2663‐2670. 24747426 10.1016/j.febslet.2014.04.012

[54] Mauviel A , Nallet‐Staub F , Varelas X . Integrating developmental signals: a Hippo in the (path)way. Oncogene. 2012;31:1743‐1756. 21874053 10.1038/onc.2011.363

[55] Zanconato F , Cordenonsi M , Piccolo S . YAP/TAZ at the roots of cancer. Cancer Cell. 2016;29:783‐803. 27300434 10.1016/j.ccell.2016.05.005 PMC6186419

[56] Zeisberg M , Neilson EG . Biomarkers for epithelial‐mesenchymal transitions. J Clin Invest. 2009;119:1429‐1437. 19487819 10.1172/JCI36183 PMC2689132

[57] Aragona M , Panciera T , Manfrin A , et al. A mechanical checkpoint controls multicellular growth through YAP/TAZ regulation by actin‐processing factors. Cell. 2013;154:1047‐1059. 23954413 10.1016/j.cell.2013.07.042

[58] Dupont S , Morsut L , Aragona M , et al. Role of YAP/TAZ in mechanotransduction. Nature. 2011;474:179‐183. 21654799 10.1038/nature10137

[59] Piersma B , Bank RA , Boersema M . Signaling in fibrosis: TGF‐β, WNT, and YAP/TAZ converge. Front Med (Lausanne). 2015;2:59. 26389119 10.3389/fmed.2015.00059 PMC4558529

[60] Liu F , Lagares D , Choi KM , et al. Mechanosignaling through YAP and TAZ drives fibroblast activation and fibrosis. Am J Physiol Lung Cell Mol Physiol. 2015;308:L344‐L357. 25502501 10.1152/ajplung.00300.2014 PMC4329470

[61] Everaerts W , Zhen X , Ghosh D , et al. Inhibition of the cation channel TRPV4 improves bladder function in mice and rats with cyclophosphamide‐induced cystitis. Proc Natl Acad Sci USA. 2010;107:19084‐19089. 20956320 10.1073/pnas.1005333107 PMC2973867

[62] Guan R , Wang X , Zhao X , et al. Emodin ameliorates bleomycin‐induced pulmonary fibrosis in rats by suppressing epithelial‐mesenchymal transition and fibroblast activation. Sci Rep. 2016;6:35696. 27774992 10.1038/srep35696 PMC5075925

[63] Szeto SG , Narimatsu M , Lu M , et al. YAP/TAZ are mechanoregulators of TGF‐β‐Smad signaling and renal fibrogenesis. J Am Soc Nephrol. 2016;27:3117‐3128. 26961347 10.1681/ASN.2015050499 PMC5042658

[64] Larue L , Bellacosa A . Epithelial‐mesenchymal transition in development and cancer: role of phosphatidylinositol 3’ kinase/AKT pathways. Oncogene. 2005;24:7443‐7454. 16288291 10.1038/sj.onc.1209091

[65] Tan WJ , Tan QY , Wang T , Lian M , Zhang L , Cheng ZS . Calpain 1 regulates TGF‐β1‐induced epithelial‐mesenchymal transition in human lung epithelial cells via PI3K/Akt signaling pathway. Am J Transl Res. 2017;9:1402‐1409. 28386365 PMC5376030

[66] Rubashkin MG , Cassereau L , Bainer R , et al. Force engages vinculin and promotes tumor progression by enhancing PI3K activation of phosphatidylinositol (3,4,5)‐triphosphate. Cancer Res. 2014;74:4597‐4611. 25183785 10.1158/0008-5472.CAN-13-3698 PMC4191931

[67] Hinz B . Tissue stiffness, latent TGF‐beta1 activation, and mechanical signal transduction: implications for the pathogenesis and treatment of fibrosis. Curr Rheumatol Rep. 2009;11:120‐126. 19296884 10.1007/s11926-009-0017-1

[68] O'Connor JW , Riley PN , Nalluri SM , Ashar PK , Gomez EW . Matrix rigidity mediates TGFβ1‐induced epithelial‐myofibroblast transition by controlling cytoskeletal organization and MRTF‐A localization. J Cell Physiol. 2015;230:1829‐1839. 25522130 10.1002/jcp.24895

[69] Matsuzaki S , Darcha C , Pouly JL , Canis M . Effects of matrix stiffness on epithelial to mesenchymal transition‐like processes of endometrial epithelial cells: implications for the pathogenesis of endometriosis. Sci Rep. 2017;7:44616. 28303918 10.1038/srep44616 PMC5356009

[70] Saito A , Nagase T . Hippo and TGF‐β interplay in the lung field. Am J Physiol Lung Cell Mol Physiol. 2015;309:L756‐L767. 26320155 10.1152/ajplung.00238.2015

[71] Xu J , Lamouille S , Derynck R . TGF‐beta‐induced epithelial to mesenchymal transition. Cell Res. 2009;19:156‐172. 19153598 10.1038/cr.2009.5 PMC4720263

[72] Valcourt U , Kowanetz M , Niimi H , Heldin CH , Moustakas A . TGF‐beta and the Smad signaling pathway support transcriptomic reprogramming during epithelial‐mesenchymal cell transition. Mol Biol Cell. 2005;16:1987‐2002. 15689496 10.1091/mbc.E04-08-0658 PMC1073677

[73] Lamouille S , Derynck R . Emergence of the phosphoinositide 3‐kinase‐Akt‐mammalian target of rapamycin axis in transforming growth factor‐β‐induced epithelial‐mesenchymal transition. Cells Tissues Organs. 2011;193:8‐22. 21041997 10.1159/000320172 PMC3030503

[74] Berridge MJ . Calcium signalling in health and disease. Biochem Biophys Res Commun. 2017;485:5. 28130105 10.1016/j.bbrc.2017.01.098

[75] Berridge MJ , Bootman MD , Roderick HL . Calcium signalling: dynamics, homeostasis and remodelling. Nat Rev Mol Cell Biol. 2003;4:517‐529. 12838335 10.1038/nrm1155

[76] Zhao B , Li L , Wang L , Wang CY , Yu J , Guan KL . Cell detachment activates the Hippo pathway via cytoskeleton reorganization to induce anoikis. Genes Dev. 2012;26(1):54‐68. 22215811 10.1101/gad.173435.111 PMC3258966

[77] Mizuno A , Matsumoto N , Imai M , Suzuki M . Impaired osmotic sensation in mice lacking TRPV4. Am J Physiol Cell Physiol. 2003;285:C96‐C101. 12777254 10.1152/ajpcell.00559.2002

[78] Zhang DX , Mendoza SA , Bubolz AH , et al. Transient receptor potential vanilloid type 4‐deficient mice exhibit impaired endothelium‐dependent relaxation induced by acetylcholine in vitro and in vivo. Hypertension. 2009;53:532‐538. 19188524 10.1161/HYPERTENSIONAHA.108.127100 PMC2694062

[79] Lichti U , Anders J , Yuspa SH . Isolation and short‐term culture of primary keratinocytes, hair follicle populations and dermal cells from newborn mice and keratinocytes from adult mice for in vitro analysis and for grafting to immune‐deficient mice. Nat Protoc. 2008;3:799‐810. 18451788 10.1038/nprot.2008.50 PMC6299324

[80] Thorneloe KS , Cheung M , Bao W , et al. An orally active TRPV4 channel blocker prevents and resolves pulmonary edema induced by heart failure. Sci Transl Med. 2012;4:159ra148. 10.1126/scitranslmed.3004276 23136043

